# Cold-Source Composite Welding Repair of 9Cr2Mo Thick-Walled Parts: Microstructure, Mechanical Properties, and Finite Element Simulation

**DOI:** 10.3390/ma17184496

**Published:** 2024-09-13

**Authors:** Danqing Yin, Haoqi Zhao, Yonglei Wang, Ning Ma, Junming Chang, Meng Wang, Jinglong Dong

**Affiliations:** 1 School of Materials Science and Engineering, Henan University of Science and Technology, Luoyang 471023, China; 220320020234@stu.haust.edu.cn (H.Z.); wylair@126.com (Y.W.); lsxzmn@163.com (N.M.); 220320020177@stu.haust.edu.cn (J.C.); 230320020230@stu.haust.edu.cn (M.W.); 2 Longmen Laboratory, Luoyang 471000, China; 3 Luoyang Leishuo Additive Manufacturing Co., Ltd., Luoyang 471100, China; 13039132526@163.com

**Keywords:** support roller, heat-affected zone, residual stress, finite element simulation, microhardness

## Abstract

The restoration of large support rollers poses an industrial challenge due to the high energy consumption of traditional repair methods. Consequently, a novel approach for repairing support rollers has been introduced and tested on thick-walled components. Finite element simulations aided the study of welding repairs for large thick-walled components, examining element distribution, microstructure, mechanical properties, and residual stress distribution across various processes. The results indicate that employing ABAQUS2023 finite element software to analyze stress variations under different working conditions, the Dynamically Controlled Low-Stress No-Distortion method effectively mitigates residual stresses both during and post welding, reducing average transverse residual stresses by 14.5% and average longitudinal residual stresses in the weld zone by 29.1%. The Dynamically Controlled Low-Stress No-Distortion method narrows the high-temperature range of the heat source, consequently decreasing the size of the heat-affected zone by 33.3% compared to conventional welding. The microstructure featured dendrites and equiaxed columnar crystals, with the Dynamically Controlled Low-Stress No-Distortion method capable of grain refinement, transforming some equiaxed columnar crystals into cellular structures. As grains were refined, microhardness improved, with the covering layer’s microhardness rising by 14.68%. A comparison between simulated and measured values of lateral and longitudinal residual stresses at corresponding points revealed discrepancies of 14.6% and 20.5% in accuracy, respectively.

## 1. Introduction

The cold roll is a key component of rolling equipment, directly operating within a steel rolling mill. Cold roll components demand excellent mechanical properties, particularly in terms of mechanical strength, necessitating a surface with uniformly distributed high hardness and wear resistance.

In practical operations, given the unique nature of the workpieces, cold rollers experience significant cyclic rolling stress [1,2], intense friction, and compression [3], leading to surface scratches [4], fractures [5], and peeling [6]. Periodic thermal cycling generates thermal stress, which, combined with rolling pressure and torque, can cause scratches on the roller surface. If these surface scratches are not promptly addressed, they can result in localized peeling under the rolling pressure. The complex alternating stresses during the rolling process can initiate cracks in areas of low surface hardness, causing them to extend in various directions. As the cracks grow, they intersect and connect, leading to the surface peeling of the roller due to the stress [7,8,9]; fractures occur when the roller experiences a large external load and becomes overloaded [10]. These forms of damage can reduce the quality and service life of the rollers, directly causing the rollers to be scrapped. The demand for cold rollers is steadily increasing, with an annual consumption of over 600,000 tons of various cold and hot rollers, causing direct economic losses exceeding CNY 15 billion, leading to substantial resource waste and environmental harm.

Various factors lead to the disposal of support rollers, many of which retain significant potential for reuse. Failing to reuse repairable spare parts results in considerable energy wastage. Previously, overlay welding was employed to restore support rollers, thereby extending their lifespan [11]. Although the rollers exhibited no signs of cracks, detachment, or other defects in the first year post repair, meeting the initial objectives, prolonged use revealed escalating issues with detachment, particularly an increase in the affected area. Secondary spalling can lead to the permanent discard of the roller, rendering it irreparable; thus, the issue of support roller detachment remains unresolved. To remedy this, we propose a repair method that manages the stress range during the repair process, aiming to limit the heat-affected zone and minimize secondary spalling.

As early as the 1960s, scholars began researching low-stress, deformation-free welding. Theoretically, by adding an auxiliary cold source to balance the heat during welding, achieving low-stress and deformation-free welding is feasible. Karlsson [12] was the first to propose using auxiliary heat sources to prevent solidification cracks in large wing plate welds. Based on this theory, scholars like Guan Qiao [13] from the Beijing Institute of Aeronautical Manufacturing Engineering developed the Low-Stress No-Distortion (LSND) welding method to prevent instability and warping in thin-walled components. This method ensures that peak residual stress after welding remains below critical instability stress levels by utilizing temperature differences created either through static temperature fields or dynamic cold sources, thus achieving the low-stress, deformation-free welding of thin plates, which has been successfully applied in aerospace thin-plate structures. American scholar Deo [14] conducted experimental research on residual stress and deformation in A36 steel conventional welded plates, testing various welding conditions including free and constrained states. Extensive experimental research revealed that auxiliary heating on either side of the centerline ahead of the heat source can induce longitudinal tensile stress in the weld structure, thus reducing residual stress and preventing post-weld warping, effectively eliminating post-weld specimen deformation.

By eliminating the dependence on the preset temperature field during welding, a specially constructed point heat sink is established, which closely tracks the welding heat source, forming a “heat source-heat sink” multi-source system to achieve a low-stress, deformation-free welding effect on the welded structure [15]. This technique is known as the Dynamically Controlled Low-Stress No-Distortion (DC-LSND) welding method. Ueda [16] employed a welding method that utilized an additional heat source followed by a heat sink for welding SUS 304 pipes of various diameters. With appropriate choices of heat sink parameters, the residual stress in the welded structure was significantly reduced.

The Harbin Institute of Technology has conducted experimental research on controlling welding cracks based on principles similar to dynamic low-stress, deformation-free welding, termed the “trailing cooling method” [17]. Extensive research and simulations have revealed that cooling contraction in the cold source region during welding causes metal compression in the preceding high-temperature area, delaying the onset of plastic tensile strain and significantly reducing the propensity for hot cracks. In subsequent studies, they employed finite element software to analyze the welded structure using the trailing cooling method, concluding that this approach reduces welding cracks and further the flexural deformation of the welded structure [18].

Preston [19] performed finite element simulations on the low-stress, deformation-free welding technique for aluminum alloys. The main focus of the simulation was to apply auxiliary heat sources on either side of the weld seam’s centerline ahead of the welding arc, ensuring they moved in sync with the arc. The findings indicate that this method significantly reduces the longitudinal welding residual stress in the structure. Michalerish [20,21] performed a finite element analysis on how the low-stress, deformation-free welding technique effectively reduces residual stress and strain in thin plates. Extensive experimental research further demonstrated that this welding method significantly lowers residual stress in welded structures, thus decreasing their instability and deformation. Referring to the principles behind the low-stress, deformation-free welding technique, Dong [22] analyzed the welding residual stress in structures with a preset temperature field for welding repair. The results closely matched experimental data, leading to the conclusion that preset temperature fields during welding repair are an effective way to reduce residual stress in welded structures. The aforementioned research primarily focuses on the welding of thin-plate structures and is not applicable to components with significant depth dimensions, and there is also limited research on stress and microstructure control in rolling mills. In Hernández-Acosta et al.’s study [23], fractional calculations were proposed to describe low-dimensional elements, integrating carbon-based thick samples, and these provided new ideas for future work.

This article introduces a novel repair method for large support rollers that circumvents traditional methods; the addition of a heat sink can compress the high-temperature influence range during the welding process, narrowing the heat-affected zone compared to traditional processes and reducing the impact on the collective. To assess the method’s feasibility, trials are initially carried out on thick-walled components. The DC-LSND method was employed with liquid nitrogen as the cold source for repairs, augmented by numerical simulations to investigate the stress distribution and temperature fluctuations under four distinct repair scenarios on 9Cr2Mo thick-walled components. Furthermore, the study examined the phase composition, microstructure, and mechanical properties, corroborating the findings through experimental validation.

## 2. Materials and Methods

### 2.1. Materials and Specimen Preparation

The substrate material used in this study is 9Cr2Mo steel, a commonly used material for Cr2 series cold rollers. Its primary applications include the manufacture of cold rollers, punches, cold stamping dies, and calendar rollers. The chemical composition is detailed in the Table 1, and the material measures 150 mm × 150 mm × 30 mm. To facilitate welding repairs, a 45 mm × 45 mm × 6 mm groove was cut at the center of the base material.

**Figure 1:**
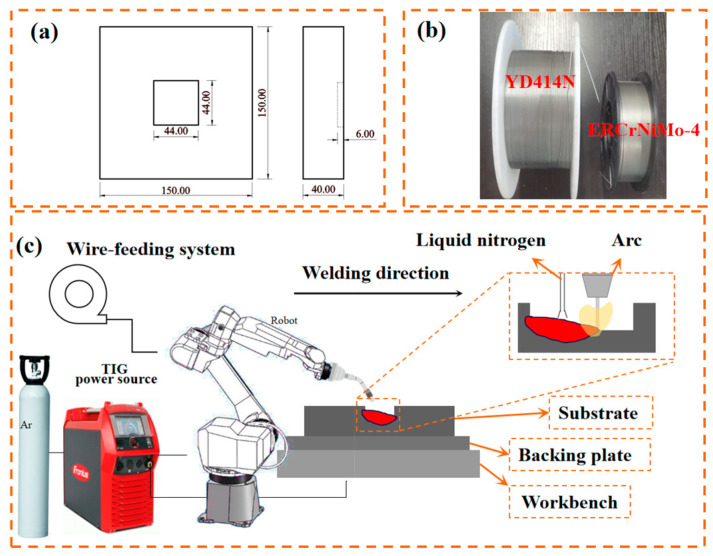
(**a**) Schematic diagram of substrate size; (**b**) welding wire morphology diagram; (**c**) working principle of TIG welding.

Prior to welding repair, the substrate is subjected to surface treatment, including grinding and sandblasting, to thoroughly remove surface oxides and impurities. Subsequently, it is cleaned with anhydrous ethanol and dried thoroughly. The material for the welding repair transition layer is ERNiCrMo-4 nickel-based alloy welding wire, 1.2 mm in diameter, primarily used for welding nickel-based alloys, dissimilar metals, nickel alloy steel, and stainless steel. It is also applicable for surfacing nickel–chromium–molybdenum alloy composite layers, with its chemical composition detailed in Table 2. Subsequently, 9Cr2Mo flux-cored welding wire YD414N, also 1.2 mm in diameter, is applied on top of the transition layer for sealing, with its chemical composition outlined in Table 3. TIG welding was selected, using a digital welder from Fronius as the arc welding power source (TIG, TPS400i, Fronius, Wels, Austria). The process flowchart is shown in Figure 1. Following initial process trials, this study ultimately identified the process parameters listed in Table 4.

### 2.2. Finite Element Model

**Figure 2:**
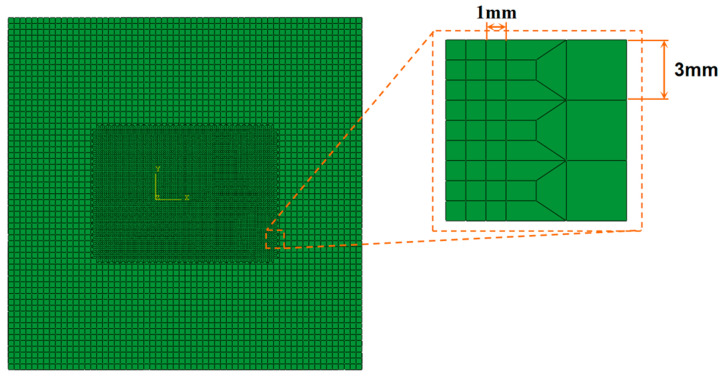
Schematic illustration of finite element model.

Selecting the appropriate mesh size is crucial in finite element simulation; a grid size that is too small can lead to excessively long computation times, and a grid size that is too large can reduce the accuracy of the calculation results [26]. To ensure simulation accuracy and enhance calculation speed, the unit size within the temperature-affected zone is set to 1 mm × 1 mm × 1 mm. In areas not affected by temperature, a 3:1 transition is applied to reduce the overall number of units and boost calculation speed. The schematic diagram of the finite element model is shown in Figure 2. The three-dimensional solid element, employing thermal displacement coupling analysis, comprises a total of 91,920 elements. The boundary conditions are fully constrained at the bottom nodes, and the initial ambient temperature is 20 °C.

The heat source employed in this study is a double ellipsoid heat source proposed by Goldak et al. [27], which involves incorporating the energy density of the bulk heat source into the node vector generated by the internal heat source. This model not only reflects the characteristics of the melt pool but also takes into account the energy distribution characteristics in the depth direction of the melt pool, resulting in a temperature field that is more in line with reality. Essentially, this model segments the heat source into anterior and posterior segments, articulated through two distinct expressions, as depicted in Formulas (1) and (2) [27].

The distribution of the volumetric heat flux density of the elliptical model in the first half is

(1) $$q\left(x,y,z\right)=\frac{6\sqrt{3}f_{1}q_{0}}{abC_{f}\pi \sqrt{\pi }}exp\left(-\frac{3x^{2}}{C_{f}^{2}}-\frac{3y^{2}}{a^{2}}-\frac{3z^{2}}{b^{2}}\right)$$

The volumetric heat flux density of the ellipse in the latter half of the model is

(2) $$q\left(x,y,z\right)=\frac{6\sqrt{3}f_{2}q_{0}}{abC_{b}\pi \sqrt{\pi }}exp\left(-\frac{3x^{2}}{C_{b}^{2}}-\frac{3y^{2}}{a^{2}}-\frac{3z^{2}}{b^{2}}\right)$$

In the formula, the letters $C_{f}$, $C_{b}$, a, and b represent shape parameters, and among them, $C_{f}$ is the length of the front half axis; $C_{b}$ is the length of the rear half axis; a is the shape parameter in the melt width direction; b is the shape parameter in the direction of the melting depth; $q_{0}$ represents effective input power; and regarding the heat source parameters, they are represented by parameters $f_{1}$ and $f_{2}$, which respectively signify the energy distribution coefficients for the elliptical spheres before and after the model. The value represented by “3” in the equation indicates the degree of concentration in heat flow distribution, which is a parameter associated with heat flow distribution.

### 2.3. Characterization

To investigate the microstructure of the coating and characterize the properties of the filler layer, samples measuring 6 mm × 6 mm × 15 mm were cut from a vertical section using an electric discharge wire-cutting machine. The samples were abraded with 180 # to 2000 # sandpaper and fine-tuned with 4000 # polishing paste to ensure consistent surface roughness. Selected cross-sectional samples underwent metallographic analysis and were etched with aqua regia (VHCL: VHNO_3_ = 3:1) for 10–15 s.

Utilize a scanning electron microscope (SEM, Crossbeam 350, Zeiss, Oberkochen, Germany) equipped with an energy-dispersive spectrometer (EDS) to analyze the macroscopic morphology, microstructure, and elemental distribution of the sample. The collected experimental data were analyzed using Oxford Channel 5. The microhardness of the coating was tested using a micro-Vickers hardness tester (HV, HV-1000TPTA, Laizhou, Shandong, China) with a 10 s loading time, a load of 0.2 KN, and an indentation spacing of 0.2 mm. We employ an X-ray residual stress analyzer (LXRD, 150PROTO, Vancouver, BC, Canada) to assess residual stresses in the weld zone and heat-affected zone. We observe the heat-affected zone with a metallographic microscope (MM, XD30M, Ningbo, China). Stress relief annealing was conducted using a muffle furnace (SGMM45/14A, sigma, Luoyang, China).

## 3. Results

### 3.1. Finite Element Simulation Results

**Figure 3:**
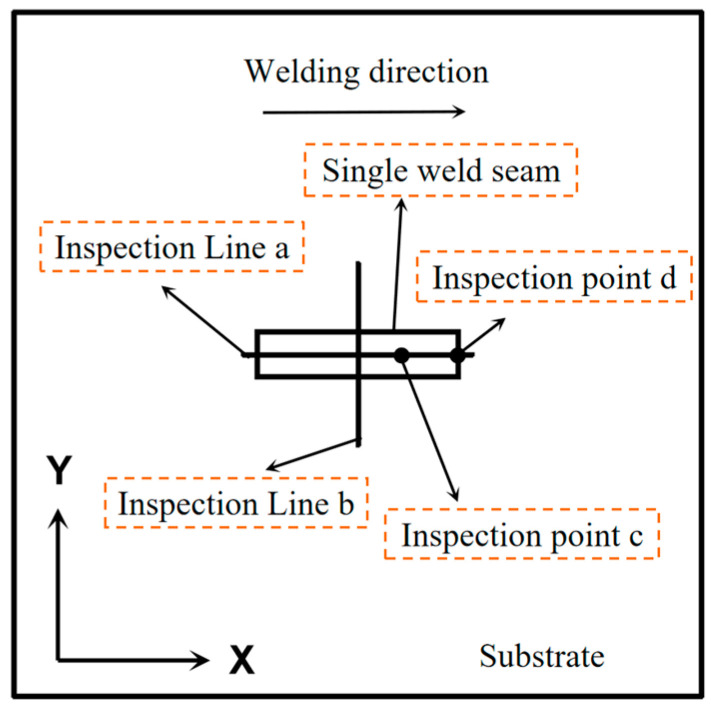
Schematic diagram of reference point selection.

To assess the method’s feasibility, three-dimensional finite element simulations were conducted. To save computation time, numerical simulations initially employed single-pass welding. We analyzed the stress distribution of four working conditions, including normal welding without any external conditions; preheating before welding; the Low-Stress No-Distortion method; and the Dynamically Controlled Low-Stress No-Distortion method. For further analysis, two inspection lines and one inspection point were selected, as depicted in Figure 3.

**Figure 4:**
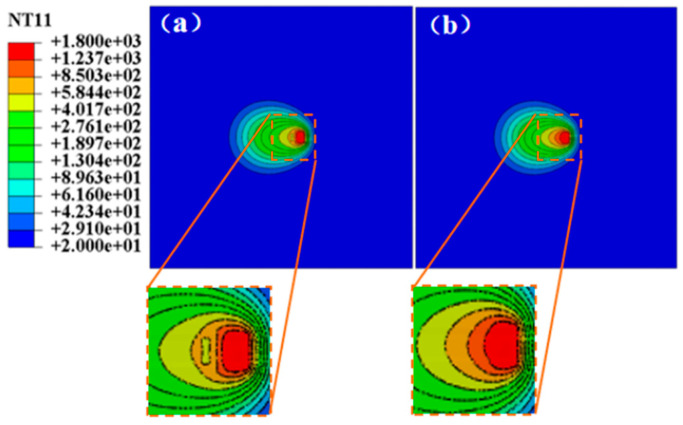
Temperature cloud map (unit: °C): (**a**) with added heat sink; (**b**) conventional welding.

At inspection point d, the temperature cloud map at the moment shows, as demonstrated in Figure 4, that the temperature field produced by DC-LSND welding is markedly distinct from that of conventional welding methods. The varying temperature hues in the cloud map indicate that, in the direction of the welding heat source’s movement, the temperature drops sharply in the heat sink region, creating a temperature valley, which represents a notable deviation from conventional welding.

Moreover, in comparison to conventional welding, the high-temperature isotherm range in the temperature field of DC-LSND welding is notably smaller than that of conventional welding. Consequently, it can be inferred that the employment of a low-stress, deformation-free welding heat sink reduces the extent of the high-temperature zone during welding. The high-temperature isotherms rapidly narrow in front of the heat sink, leading to a marked difference in isotherm shape from the typical ellipses seen in conventional isotherms. Owing to the heat sink’s rapid cooling effect, the isotherms are sharply concentrated in front of it, causing a substantial temperature gradient between the heat source and the heat sink.

**Figure 5:**
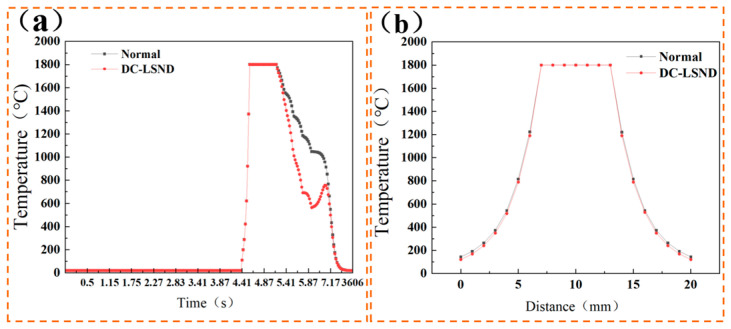
(**a**) Temperature-time curve at reference point c; (**b**) temperature at node b of reference line.

We obtain the temperature–time curves for the DC-LSND method and conventional welding at point c. From Figure 5a, it can be seen that the temperature remains at 20 °C for 0 to 4.4 s. The temperature starts to rise as the heat source approaches, peaks when the weld reaches point c, and then cools as the heat source recedes. In the welding process with a heat sink, the temperature plummets due to the heat sink’s rapid cooling effect. The temperature in front of the heat sink is 692 °C compared to about 1186 °C in conventional welding at the same location. After the heat sink passes, the welded structure’s temperature rises, primarily from the surrounding high-temperature metal, though it remains significantly lower than in conventional welding. Figure 5b shows that adding a heat sink lowers the heat-affected zone’s temperature by 20 °C at the same point, further reducing the extent of its effects and the base material’s phase transformation due to temperature changes.

The Huber–Von Mises yield criterion is a critical parameter for assessing weld integrity and the presence of cracks, and it was first proposed by Huber [28] in 1904 and perfected by von Mises [29] in 1913. Its fundamental principle posits that materials yield at a specific point when the cumulative elastic strain energy per unit volume reaches a defined threshold. As depicted in Formula (3), this criterion introduces the concept of equivalent stress, which is independent of the material’s specific stress state, through an analysis of the principal stress. The critical step in determining whether a material has entered the plastic deformation stage involves comparing this equivalent stress with the material’s yield strength. Once the material enters the plastic state, its equivalent stress demonstrates a stable and unchanging characteristic [30].

(3) $$\bar{\sigma }=\frac{\sqrt{2}}{2}\sqrt{{\left(\sigma_{1}-\sigma_{2}\right)}^{2}+{\left(\sigma_{2}-\sigma_{3}\right)}^{2}+{\left(\sigma_{3}-\sigma_{1}\right)}^{2}}\leq \sigma_{s}$$

In this formula, $\bar{\sigma }$ denotes equivalent stress, while $\sigma_{1},\sigma_{2}$, and $\sigma_{3}$ correspond to the principal stress components in the three mutually orthogonal directions of the Cartesian coordinate system (X, Y, and Z axes), respectively; $\sigma_{s}$ denotes the critical stress value at which the material yields, namely the yield limit.

**Figure 6:**
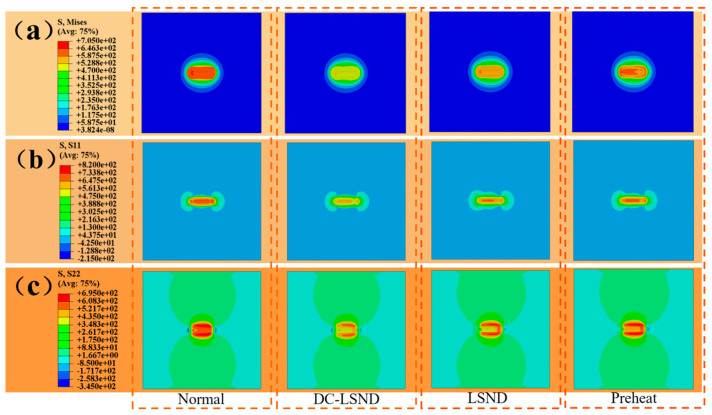
Stress cloud map under different working conditions (unit: MPa), and the dashed box represents different stresses under the same working condition: (**a**) Huber-Mises stress; (**b**) lateral residual stress; (**c**) longitudinal residual stress.

The Figure 6 displays stress comparison charts for various working conditions. The charts reveal substantial residual stresses in and around the weld area. Conventional welding results in the highest residual stress, whereas the DC-LSND method yields the lowest. Significant transverse tensile stress occurs in the weld seam area, and considerable longitudinal tensile stress arises near the weld seam in the parallel welding direction.

During the conventional welding process, points both within the weld seam and those nearby undergo compressive plastic deformation. Upon the arrival of the welding heat source, the metal in these areas begins to melt, creating a molten pool. Although the molten metal is stress- and strain-free, it melts within the context of the nearby deformed region. After the heat source moves on, the metal in the weld pool starts to solidify, forming a weld seam. As it cools, the weld seam rapidly solidifies, while the metal near the seam cools more slowly due to the transfer of heat from the weld seam. This differential cooling causes the metal in the weld seam to be stretched by the metal near it [31].

In DC-LSND welding, the heat sink’s rapid cooling shrinkage effect on the metal in the weld area induces strong tensile stress in the high-temperature metal between the melt pool and the heat sink. This leads to heightened tensile stress in the metal both within the weld area and adjacent to it during the cooling process. Consequently, this reduces the coordinated strain and lowers the residual stress post welding.

**Figure 7:**
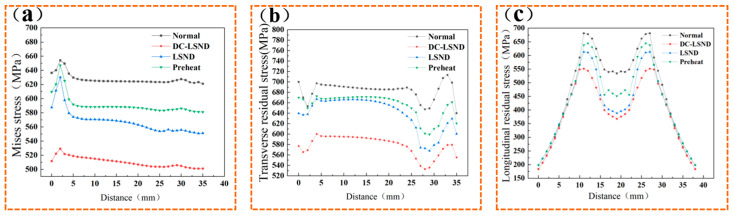
Stress curves of nodes under different operating conditions: (**a**) investigation of Huber-Mises stress on line a; (**b**) investigation of lateral residual stress on line a; (**c**) investigation of longitudinal residual stress on line b.

Figure 7a illustrates the Huber-Mises stress distribution at each node along inspection line a. Under all four conditions, high stress arises at the substrate’s initial position upon commencing welding. This arises because the welding’s initiation site is the first to absorb heat during the process. With the influx of heat, materials undergo thermal expansion. At the outset of welding, considerable heat accumulation in this region leads to localized overheating and substantial thermal stress. As the welding continues, heat will spread outward, cooling the initial high-temperature zone, yet the accumulated stress may remain considerable.

Figure 7b illustrates the distribution of lateral residual stress at each node along inspection line a. Under all four conditions, stress troughs form at the substrate’s starting and ending positions during welding. This occurs because the rapid cooling of the weld seam and slower cooling in the heat-affected zone during welding generate substantial tensile stress, which counteracts some of the tensile stress in the region.

Figure 7c illustrates the longitudinal residual stress at each node along investigation line b. The figure reveals a symmetrical distribution of longitudinal residual stress along the weld seam’s centerline. The longitudinal residual stress within the weld zone is significantly lower than in the adjacent areas. This is attributed to the higher internal stress generated in the adjacent areas due to the weld seam’s temperature, which is subsequently released during rapid cooling. The metal adjacent to the weld seam cools more slowly due to the transfer of weld temperature, preventing the full release of residual stresses. LSND and DC-LSND are employed due to their provision of an external cooling source to the weld zone, ensuring uniform cooling. Other methods result in stress fluctuations in the weld zone due to thermal convection effects.

Table 5 clearly shows that DC-LSND effectively reduces residual stresses after welding. The average Huber–Mises stress in the weld zone was reduced by 18.6%, the average transverse residual stress decreased by 14.5%, and the average longitudinal residual stress dropped by 29.1%.

To conduct a more comprehensive analysis of stress variations during the welding process, stress cloud maps were selected from the initial 2 s, 4 s, and 6 s of welding.

**Figure 8:**
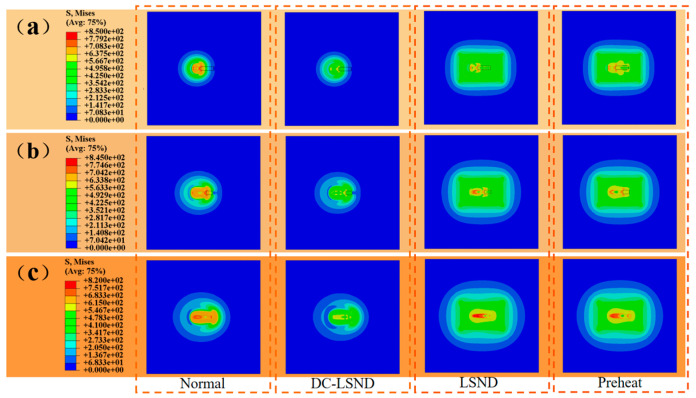
Residual stress cloud map of different welding methods at different times (unit: MPa): (**a**) Huber-Mises stress; (**b**) lateral residual stress; (**c**) longitudinal residual stress.

Figure 8 displays the residual stress cloud maps for various welding methods at different times. As illustrated, DC-LSND effectively mitigates residual stress throughout the welding process. Introducing preheating along with LSND reduces residual stress in the weld seam during the initial stage; however, this results in substantial stress in the heating zone. As the welding progresses, a significant stress concentration forms at the starting position of the weld seam, which is subsequently released during the cooling process [32].

**Figure 9:**
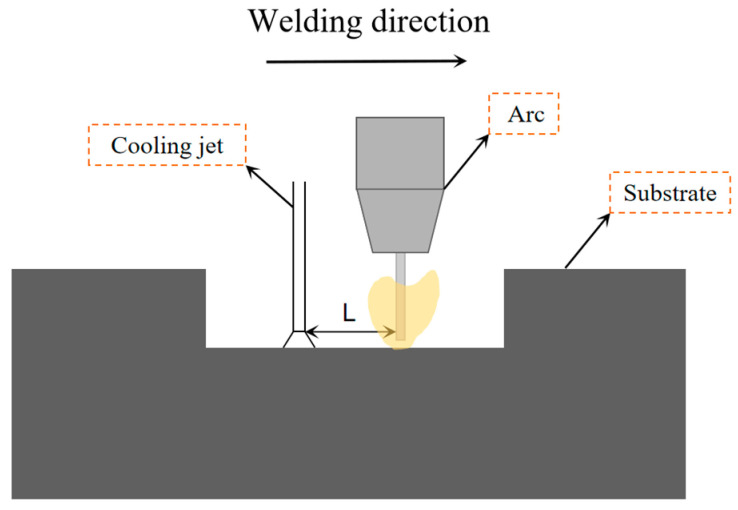
Schematic diagram of distance L between heat source and heat sink.

Compared to conventional welding methods, DC-LSND welding technology alters the stress–strain distribution in welded joints. However, it is essential to choose the process parameters correctly. If the welding parameters are incorrectly selected, low-stress deformation-free welding may not only fail to achieve the desired results but could even worsen the outcome. Therefore, the influence of distance L between the heat source and the heat sink is studied and analyzed. A schematic diagram of the distance between the heat source and the heat sink is shown in Figure 9.

The final stress distribution was investigated for distances L between the heat source and the heat sink at 3 mm, 7 mm, 11 mm, 15 mm, and 20 mm, respectively. Among them, 3 mm is the result calculated previously.

**Figure 10:**
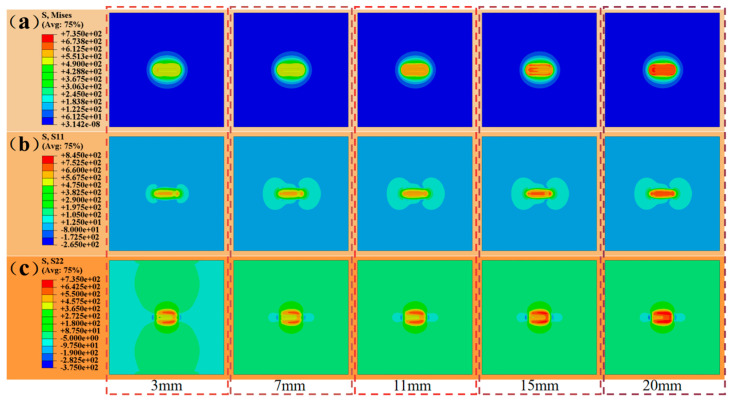
Stress cloud maps at different distances L (unit: MPa): (**a**) Huber-Mises stress; (**b**) lateral residual stress; (**c**) longitudinal residual stress.

The figure displays stress comparison charts for various distances, with Figure 10a being the Huber-Mises stress cloud map. The graph indicates that the residual stress values in the weld zone and near the weld zone increase with greater distances. Figure 10b depicts the transverse residual stress cloud map at different distances, resulting in considerable tensile stress in the weld zone that intensifies with distance. Figure 10c shows the longitudinal residual stress cloud map under different process conditions, resulting in significant tensile stress near the weld seam in the parallel welding direction and compressive stress at both weld seam ends.

**Figure 11:**
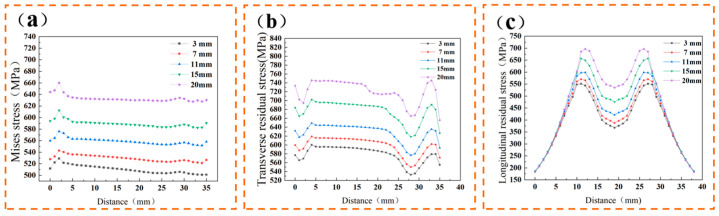
Node stress at different distances L: (**a**) investigating the Huber-Mises stress of nodes on line A; (**b**) investigating the lateral residual stress of nodes on line A; (**c**) investigating the longitudinal residual stress of nodes on line B.

Figure 11a compares the Huber-Mises stress at each node along line a at various distances. The figure demonstrates that as the distance between the heat source and the heat sink increases, so does the residual stress. This is because the weld seam has already cooled to a lower temperature when the heat sink enters, and the addition of strong cooling at this point generates substantial internal stress within the weld seam. After the heat sink leaves, the temperature of the weld seam drops further, preventing the internal stress from effectively releasing with the drop in temperature. In actual experiments, positioning the heat sink too close to the weld seam can compromise the heat source’s stability.

Figure 11b illustrates the lateral residual stress distribution at each node along inspection line a. As the distance between the heat sink and the heat source increases, the lateral residual stress also rises. Stress minima are observed at the substrate’s initial and final welding positions. This is due to the rapid cooling of the weld seam and the slower cooling in the heat-affected zone, which induce substantial tensile stress in the weld seam, balancing out some of the tensile stress in this region. Due to the welding process, the temperature is transmitted to the substrate in front of the welding direction, increasing the substrate temperature and reducing the temperature difference, resulting in a decrease in stress at the end of the welding.

Figure 11c illustrates the longitudinal residual stresses at each node along line b. The figure reveals a symmetrical distribution of longitudinal residual stresses along the centerline of the weld seam. The longitudinal residual stress in the weld zone is significantly lower than in the adjacent areas. This is attributed to the higher internal stress generated in the adjacent areas due to the temperature effects of welding, which is subsequently released during the rapid cooling of the weld. The metal adjacent to the weld cools more slowly due to the heat transfer from the weld, preventing the full relaxation of the residual stresses.

Table 6 clearly shows that with increasing distance, the residual stress in the weld zone also rises. In practical operations, an excessively close distance between the heat source and the heat sink can compromise the heat source’s stability, subsequently impacting the molding results. Hence, an optimal distance of L = 7 mm is determined.

Using optimized parameters for multi pass welding simulation, with a 50% overlap rate, a weld width of 10 mm, and a pit width of 44 mm, there are a total of eight weld seams.

**Figure 12:**
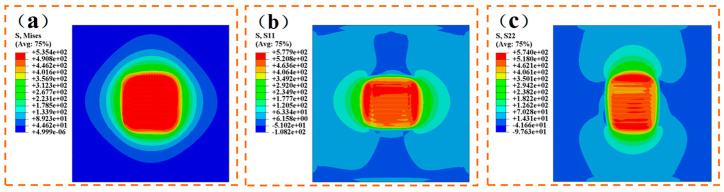
Multi pass welding stress cloud map (unit: MPa): (**a**) Huber-Mises stress; (**b**) lateral residual stress; (**c**) longitudinal residual stress.

Figure 12a presents the Huber-Mises stress cloud map, revealing a uniform stress distribution within the weld zone. The average stress across all nodes in the weld zone is 505.4 MPa, ranging from a maximum of 510.2 MPa to a minimum of 492.6 MPa. Figure 12b displays the transverse residual stress cloud map. The average stress across all nodes in the weld zone is 517.4 MPa, ranging from a maximum of 547.5 MPa to a minimum of 392.2 MPa. Figure 12c illustrates the longitudinal residual stress cloud map, with an average stress across all nodes in the weld zone of 521.7 MPa. The maximum stress reaches 533.5 MPa, while the minimum stands at 439.3 MPa.

Figure 12 shows that significant transverse residual stresses are induced in the base metal perpendicular to the welding direction. As the welding progresses, these residual stresses accumulate, reaching peak levels in the final two welds. Meanwhile, the base metal aligned with the welding direction experiences pronounced longitudinal residual stresses. This is attributed to stress buildup in the material near the weld seam, which slows down due to heat from the weld, resulting in a delayed cooling rate.

**Figure 13:**
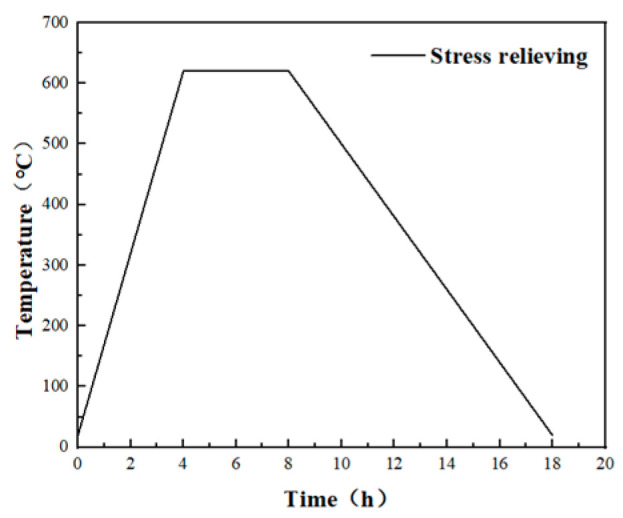
Stress relief annealing process flow chart curve.

To reduce stress, the welded workpiece undergoes stress relief annealing, as depicted in Figure 13. The heating rate is 150 °C/h, with the temperature raised to 620 °C, held for 4 h, and then cooled to room temperature in the furnace for 10 h.

**Figure 14:**
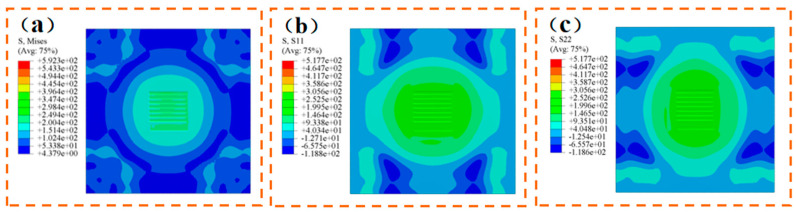
Stress cloud map after stress relief annealing: (**a**) Huber-Mises stress; (**b**) lateral residual stress; (**c**) longitudinal residual stress.

As shown in Figure 14, the stress in the weld zone notably reduces after stress relief annealing, with a uniform distribution. The average Mises stress for each node in the weld zone is 235.5 MPa. The average horizontal residual stress is 213.9 MPa, and the average vertical residual stress is 233.1 MPa.

### 3.2. Morphology and Microstructure

**Figure 15:**
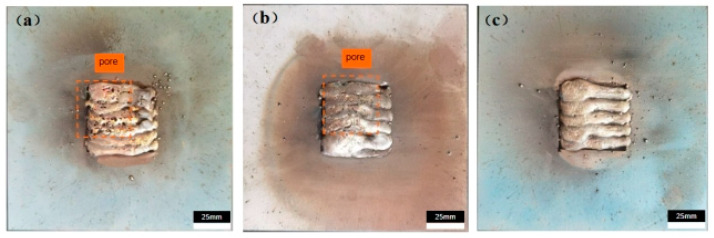
Macroscopic morphology under different working conditions: (**a**) normal welding; (**b**) preheating; (**c**) DC-LSND.

Figure 15a depicts normal welding, revealing numerous holes in the final welds and bulging at their ends. This is attributed to excessive temperature accumulation. During the heating of flux-cored wire, the core material expands, generating gas. If the temperature is excessively high, the gas cannot be released after the core material melts during welding, leading to porosity. Bulging results from excessive gas production that cannot escape quickly enough. At this stage, the weld seam has not fully solidified, and the liquid metal’s fluidity is poor post welding. Consequently, the residual gas forces its way through the liquid metal, causing it to overflow.

Figure 15b illustrates the preheating process around the weld seam prior to welding. Initially, the temperature surrounding the weld seam is consistently maintained at approximately 200 °C. Regulating the temperature throughout the welding process can minimize heat transfer, ensuring a thorough release of gasses evolved from the molten core material. The figure reveals indentations on the surface of the final welds, attributed to an abundance of water molecules in the air during a prolonged local rainstorm.

Figure 15c illustrates welding performed using the dynamic low-stress, deformation-free method, revealing a weld seam surface devoid of porosity. The application of liquid nitrogen enables the rapid cooling of the weld seam, and its spraying promotes molten pool circulation, aiding in gas evolution. Additionally, liquid nitrogen serves as a protective shield, minimizing the effects of atmospheric water molecules on the weld seam.

**Figure 16:**
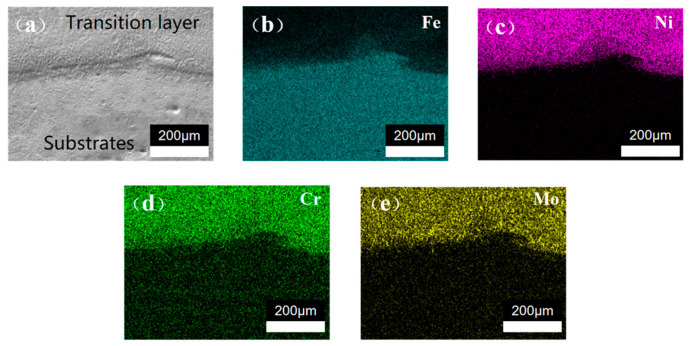
EDS analysis of matrix and transition layer: (**a**) SEM image; (**b**) Fe element distribution; (**c**) Ni element distribution; (**d**) Cr element distribution; (**e**) Mo element distribution.

**Figure 17:**
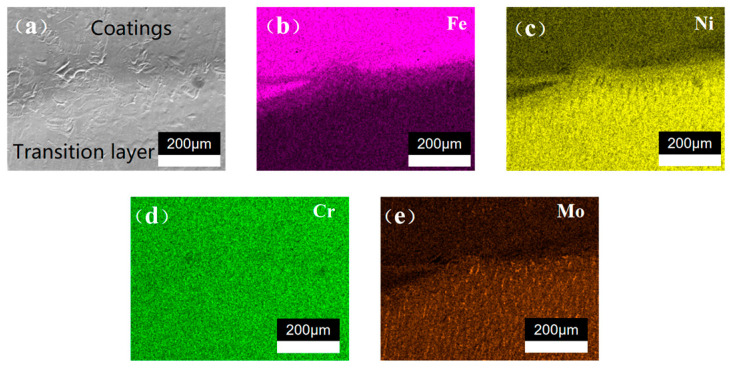
EDS analysis of transition layer and cover layer: (**a**) SEM image; (**b**) Fe element distribution; (**c**) Ni element distribution; (**d**) Cr element distribution; (**e**) Mo element distribution.

To delve deeper into the interlayer bonding, an EDS analysis was conducted on the DC-LSND sample, as illustrated in Figure 16 and Figure 17. It can be seen from the figure that strong adhesion between the substrate and transition layer occurs, as well as between the transition layer and the cover layer. As shown in Figure 16a and Figure 17a, a distinct boundary is evident between the layers. Figure 16b–e and Figure 17b–e demonstrate that the element distribution in each layer aligns with its chemical composition, showing uniform distribution. Near the boundary, minor element segregation occurs, attributed to dilution during welding.

**Figure 18:**
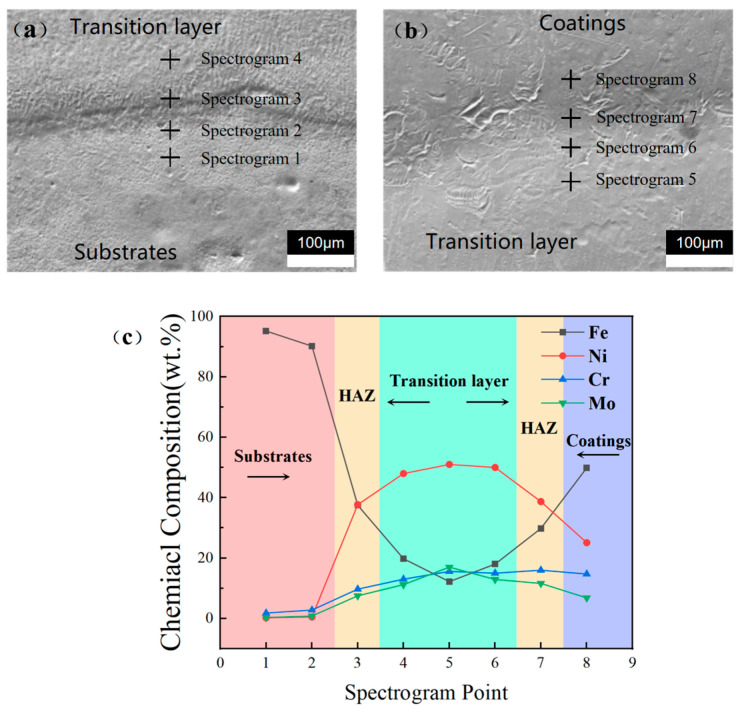
Schematic diagram of point scanning position: (**a**) matrix and transition layer; (**b**) transition layer and cover layer; (**c**) spectrogram point element composition distribution.

Point scanning reveals the elements’ segregation, as illustrated in Figure 18. As shown in Figure 18c, elements near the boundary are segregated, and the composition in the HAZ varies to different extents.

**Figure 19:**
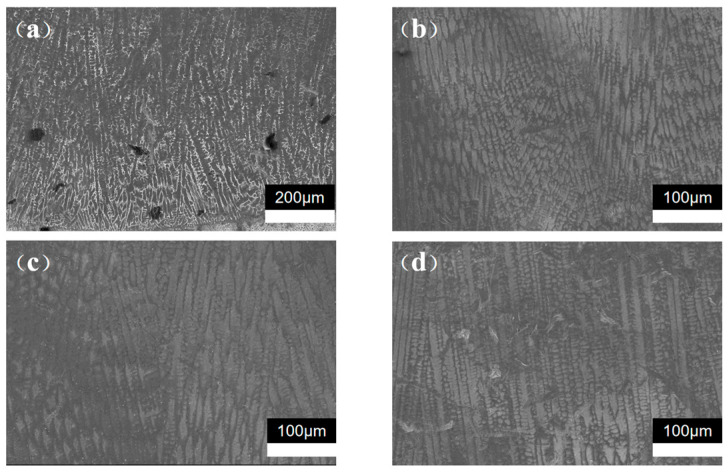
SEM image: (**a**) transition layer bottom; (**b**) cover layer bottom; (**c**) cover layer top; (**d**) DC-LSND cover layer top.

To further study the material’s microstructure, SEM analysis was conducted on corroded samples. The SEM image taken along the vertical cross-section is displayed. As shown in Figure 19a, the grains within the transition layer primarily consist of dendrites, equiaxed columnar crystals, and a small amount of fine grains at the interfaces. Figure 19b,c reveal that the covering layer is predominantly composed of equiaxed columnar crystals and dendrites. The morphology of the grains is primarily influenced by variations in the temperature gradient (G) and cooling rate (R). When the molten pool makes contact with the substrate, fine grains initially form at the interfaces due to the significant temperature gradient. As the molten pool’s heat rapidly transfers to the substrate, the temperature differential between the molten pool and the base material diminishes, leading to the formation of equiaxed columnar crystals and dendrites within the molten pool, nucleated around the fine grains. As seen in Figure 19d, the addition of liquid nitrogen transforms some equiaxed columnar crystals and dendrites into cellular crystals. This transformation occurs because the introduction of liquid nitrogen at the top of the molten pool enhances the cooling rate, preventing the columnar crystals from growing into individual cellular structures.

**Figure 20:**
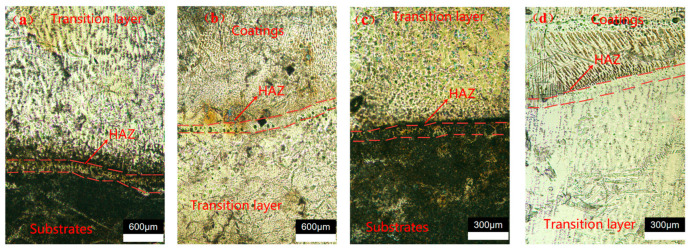
HAZ range under metallographic microscope: (**a**) transition zone between conventional welding substrate and intermediate layer; (**b**) transition zone between conventional welding intermediate layer and cover layer; (**c**) transition zone between DC-LSND substrate and intermediate layer; (**d**) transition zone between DC-LSND intermediate layer and cover layer.

To further study the effects of liquid nitrogen, the morphology of the heat-affected zones in conventional welding and DC-LSND was examined under a metallographic microscope. Figure 20a,b show that the heat-affected zone in conventional welding is about 0.15 mm, while Figure 20c,d indicate that in DC-LSND, it is about 0.1 mm. The heat-affected zone in DC-LSND is 33.3% smaller than that in conventional welding.

### 3.3. Microhardness and Residual Stress

**Figure 21:**
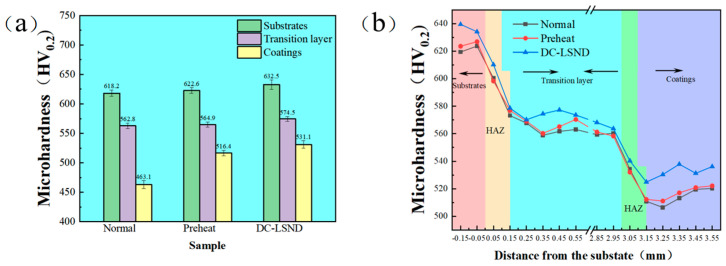
Microhardness: (**a**) average microhardness; (**b**) microhardness distribution diagram.

Vickers microhardness measurements were taken every 0.1mm from the substrate, with the average hardness values presented in Table 7. The error bar is obtained by calculating the standard deviation. In comparison to conventional welding, the use of preheating boosted the hardness by 0.71%, 0.37%, and 11.51%, respectively. The hardness achieved through the DC-LSND method increased by 2.31%, 2.08%, and 14.68%, respectively. As illustrated in Figure 21, the DC-LSND method enhances the coating’s hardness. This enhancement is attributed to the grain refinement and strengthening induced by the liquid nitrogen addition, which increases the grain boundary area per unit volume, thereby impeding dislocation movement and enhancing hardness [33].

We utilize LXRD to assess the transverse and longitudinal residual stresses following stress relief annealing. For comparative purposes, we select points along the centerline, with one point every 5 mm in the weld zone and one point each in the base and HAZ. The error bar is obtained by calculating the standard deviation.

**Figure 22:**
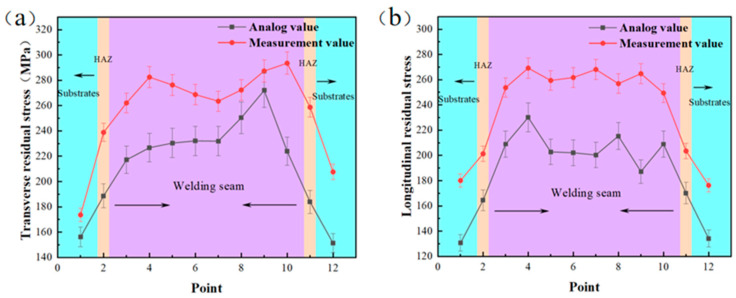
Comparison of residual stress between finite element numerical simulation values and measured values: (**a**) lateral residual stress; (**b**) longitudinal residual stress.

Figure 22 indicates that the measured values exceed the simulated values, both representing tensile stresses. The average lateral residual stress for the measured values is 275.8 MPa, while the average longitudinal residual stress is 260.4 MPa. For the same points, the simulated values exhibit an average lateral residual stress of 235.6 MPa and an average longitudinal residual stress of 207.1 MPa. The accuracy of the simulated values compared to the measured values varies by 14.6% and 20.5%, respectively.

## 4. Conclusions

Various welding repair methods were compared using finite element numerical simulations. Ultimately, the DC-LSND method was selected for the welding repair of large thick-walled components, and an investigation was conducted on the microstructure, microhardness, and residual stress properties post welding. The conclusions of the study are as follows:

(1) The finite element simulation results indicate that the DC-LSND method is also suitable for thick components. It effectively reduces residual stress both during and post welding. The inclusion of a cold source narrows the high-temperature range of the heat source, and the high-temperature isotherms rapidly contract in front of the heat sink, leading to a marked deviation in isotherm shape from the typical elliptical form. Due to the heat sink’s rapid cooling effect, the isotherms are highly concentrated in front of it, creating a substantial temperature gradient between the heat source and the heat sink. DC-LSND effectively reduces residual stresses after welding. The average Mises stress in the weld zone was reduced by 18.6%, the average transverse residual stress decreased by 14.5%, and the average longitudinal residual stress dropped by 29.1%.
(2) The DC-LSND method effectively reduces weld porosity and enhances grain refinement. Observations under a light microscope indicate that the heat-affected zone for the DC-LSND method is 33.3% smaller than that of other methods. This is attributed to the dilution rate, which results in minimal elemental segregation between layers.
(3) The microhardness of the DC-LSND method has increased primarily because of fine-grain strengthening. The residual stress after annealing was measured using LXRD, and the accuracy of the simulated and measured values differed by 14.6% and 20.5%, respectively.

## Figures and Tables

**Table 1:** Chemical composition of 9Cr2Mo Steel [24].

C	Si	Mn	P	S	Cr	Ni	Mo	Fe
0.85–0.95	0.25–0.45	0.2–0.35	0.025	0.025	1.7–2.1	≤0.25	0.2–0.4	Bal.

**Table 2:** Chemical composition of ERNiCrMo-4 [25].

C	Mo	W	Cr	Fe	Mn	Si	Ni
0.01	16.1	3.65	15.55	5.5	0.55	0.04	Bal.

**Table 3:** Chemical composition of YD414N.

C	Si	Mn	Cr	Mo	Ni	Fe
0.07	0.65	1.45	12.51	1.15	>3	Bal.

**Table 4:** TIG process parameters.

Parameter	Values	
Sample	ERNiCrMo-4	YD414N
Wire feeding speed (m/min)	7.5	7.5
Welding speed (m/min)	60	50
Elongation of welding wire (mm)	10	12
Current (A)	191	188
Voltage (V)	22.7	21.9
Protective gas flow rate (L/min)	10	10
Overlap rate (%)	50%	50%

**Table 5:** Maximum stress and average stress under different operating conditions.

Welding Conditions	Normal	DC-LSND	LSND	Preheat
Maximum Huber-Mises stress/MPa	703.5	549.6	630.0	667.2
Average Huber-Mises stress/MPa	626.2	510.0	565.2	588.5
Maximum lateral residual stress/MPa	699.9	600.1	667.1	672.7
Average lateral residual stress/MPa	689.1	590.7	661.3	668.1
Maximum longitudinal residual stress/MPa	692.6	600.7	664.8	683.1
Average longitudinal residual stress/MPa	541.7	384.2	402.6	461.3

**Table 6:** Maximum and average stress at different distances L.

Distance L/mm	3	7	11	15	20
Maximum Huber-Mises stress/MPa	549.6	575.1	612.5	677.3	732.1
Average Huber-Mises stress/MPa	510.0	529.6	558.7	608.5	656.7
Maximum lateral residual stress/MPa	600.1	618.9	649.2	702.1	745.7
Average lateral residual stress/MPa	590.9	611.1	640.8	691.7	732.3
Maximum longitudinal residual stress/MPa	551.4	570.6	598.5	657.1	696.8
Average longitudinal residual stress/MPa	384.2	404.1	436.1	489.7	551.1

**Table 7:** Average microhardness values.

Sample	Substrates (HV)	Transition Layer (HV)	Coatings (HV)
Normal	618.2 ± 5.2	562.8 ± 4.6	463.1 ± 6.6
Preheat	622.6 ± 4.9	564.9 ± 4.5	516.4 ± 4.9
DC-LSND	632.5 ± 8.1	574.5 ± 4.1	531.1 ± 6.5

## Data Availability

The data presented in this study are available on request from the corresponding author. The data are not publicly available due to privacy reasons.
